# Comparison of self-collected and healthcare worker-collected rectovaginal swabs for group B streptococcus detection in pregnancy using PCR with a commercial collection-enrichment device

**DOI:** 10.3389/fmicb.2025.1541319

**Published:** 2025-02-05

**Authors:** Iva Kukovica, Neža Omahen, Nika Klobučar, Martina Bučar, Anita Franko Rutar, Tina Perme, Miha Lučovnik, Samo Jeverica

**Affiliations:** ^1^Department of Perinatology, University Medical Centre Ljubljana, Ljubljana, Slovenia; ^2^National Laboratory of Health, Environment and Food, Maribor, Slovenia; ^3^Community Health Center Novo mesto, Novo mesto, Slovenia; ^4^Community Health Center Trebnje, Trebnje, Slovenia; ^5^Medical Faculty, University of Ljubljana, Ljubljana, Slovenia; ^6^Izola General Hospital, Izola, Slovenia

**Keywords:** group B streptococcus, self-collection, enrichment culture, PCR, chromogenic agar, screening, pregnancy

## Abstract

**Introduction:**

Universal screening for the detection of group B streptococcus (GBS) colonization in pregnant women was recently introduced in Slovenia. The aim of our study was to determine whether self-collection of rectovaginal swabs is a valid alternative to collection by healthcare workers (HCWs).

**Methods:**

A prospective, multicenter study was conducted between June and November 2023. A total of 227 pregnant women (aged 20 to 44 years) from the University Medical Center Ljubljana (*n* = 136), the Novo mesto Community Health Center (*n* = 48) and the Trebnje Community Health Center (*n* = 43) were included. Two swabs were taken: swab A by the HCWs using standard semi-solid Amies transport medium (Meus; current standard) and swab B by the pregnant woman following visual instructions using a commercial LIM Broth (Copan). Swabs were inoculated onto ChromID Strepto B (STRB) agars directly and after overnight enrichment in LIM broth. The NeuMoDx GBS assay was performed from the enrichment broth. A self-assessment questionnaire was completed after sampling. Performance characteristics were calculated and compared between different diagnostics test algorithms using McNemar’s test for paired samples.

**Results:**

Overall, GBS was detected in 18% (95% CI 13–23%; *n* = 40) of swabs A and 19% (95% CI 14–25%; *n* = 43) of swabs B. PCR was superior in both groups. In the group of swabs collected by HCWs, 4 (40 vs. 36; 11.1% difference; *p* = 0.046) and 3 (40 vs. 37; 8.1% difference; *p* = 0.083) additional positives were detected with PCR compared to direct and enrichment culture, respectively; in the group of self-collected swabs, 4 (43 vs. 39; 10.3% difference; *p* = 0.046) and 6 (43 vs. 36; 16.2% difference; *p* = 0.014) additional positives were detected with PCR compared to direct and enrichment culture, respectively. Self-collection showed a trend towards a higher diagnostic yield. PCR after enrichment from self-collected samples was found to be the most sensitive method overall. 58.5% (*n* = 124/212; 95% CI 52–65%) of women would prefer the swabs taken by HCWs.

**Discussion:**

Self-collection of rectovaginal swabs during pregnancy is a good alternative to HCW-collected swabs. PCR from enrichment broth was better for the detection of GBS compared to enrichment culture. Majority of women preferred swabs taken by HCWs.

## 1 Introduction

Invasive neonatal infections (i.e., sepsis, meningitis and pneumonia) are a major cause of morbidity and mortality in newborns worldwide and can have long-term health consequences for the child, despite appropriate treatment. Group B streptococcus (GBS) is the leading causative agent, responsible for up to 50% of invasive infections in this population (Madrid et al., 2017). GBS infections are categorized as early-onset (0–7 days of age) and late-onset (8–90 days of age). Early-onset neonatal infections are most commonly the result of vertical transmission of the pathogen from mother to newborn during childbirth, while the majority of late-onset neonatal infections are due to transmission of the pathogen through caregivers and environmental contact (Cortese et al., 2016). Before the introduction of prevention strategies, the incidence of early-onset GBS infections was 2–3 times higher than the incidence of late-onset GBS infections, and this is still the case to some extent in areas before the systematic introduction of prevention strategies (Verani et al., 2010; Lasič et al., 2018). Prevention of early-onset GBS is possible with intrapartum antibiotic prophylaxis (IAP) and is most effective when IAP is administered based on the GBS colonization status of the pregnant woman in the late third trimester of pregnancy, usually between the 35–37 weeks of gestation (Boyer et al., 1983; Boyer and Gotoff, 1986; Verani et al., 2010). Penicillin is the drug of choice for IAP as GBS remains almost universally susceptible to this antibiotic. Penicillin-allergic women require alternative agents such as cefazolin, clindamycin or vancomycin, which can be used depending on the type of allergic reaction to penicillin and the susceptibility phenotype of the GBS (Verani et al., 2010). Approximately 15–20% of pregnant women are colonized with GBS worldwide (Russell et al., 2017).

In Slovenia, invasive infections are responsible for about one fifth of neonatal deaths. The incidence of neonatal GBS infections was estimated at 0.53 per 1,000 births, which is more than 30% higher than the global and European average (Edmond et al., 2012; Lasič et al., 2018). In addition, the prevalence of colonization in pregnant women was 17.1%, and the most recent national study found that 52% of affected infants had no perinatal risk factors, confirming the inadequacy of GBS prevention based solely on the presence of risk factors during pregnancy (Lasič et al., 2018; Lučovnik et al., 2016). In 2019 the Health Council of the Republic of Slovenia adopted a proposal to introduce universal screening for GBS at 35 to 37 weeks of gestation using the PCR-based enrichment culture approach, however, due to COVID-19 pandemic it was not enacted until 2023 (Official Gazette of the Republic of Slovenia, 2023).

The collection of rectovaginal swabs for GBS colonization detection is traditionally performed by healthcare workers (HCWs). However, due to resource limitations and broader societal changes, particularly following the COVID-19 pandemic, self-collection has emerged as a complementary strategy in prevention strategies of many infection diseases as it offers improved accessibility, convenience, and privacy, which may enhance compliance with screening programs (Arbyn et al., 2014; Smith et al., 2024; World Health Organization, 2024). Additionally, collection devices have undergone significant advancements recently, replacing traditional transport media with enrichment broth. This modification enables the enrichment process to begin immediately after sample collection and, to some extent, even during transport under suboptimal conditions for bacterial growth. These devices can also streamline laboratory workflows by eliminating the need to inoculate enrichment broth and can be utilized directly for downstream molecular testing. While existing studies suggest that HCW-collected samples achieve diagnostic accuracy comparable to self-sampling, data on the performance of combined collection-enrichment devices are limited (The American College of Obstetricians and Gynecologists, 2020; Odubamowo et al., 2023). Moreover, no pragmatic studies have directly compared these two approaches.

The aims of our study were to (a) assess the diagnostic performance of self-collected and HCW-collected rectovaginal swabs for detecting GBS colonization in pregnant women at 35–37 weeks of gestation, using different diagnostic protocols: direct culture, enrichment culture, and PCR from overnight enrichment; and (b) evaluate participant feedback on the self-collection protocol in our clinical setting.

## 2 Methods

### 2.1 Clinical setting and sample collection

We conducted a prospective cohort, multicenter study between June and November 2023. Three gynecology and obstetrics departments were included in the study. The University Medical Center Ljubljana (UMC Ljubljana) in the Central Slovenia region, which is a tertiary care teaching hospital that also offers regular outpatient programs for pregnant women. The Novo mesto Community Health Center (CHC Novo mesto) and the Trebnje Community Health Center (CHC Trebnje), both located in the Southeast Slovenia region, are primary care facilities. All centers are part of the public healthcare system in Slovenia and were included in the study as a major prenatal healthcare providers of the two regions. Screening for GBS colonization is part of routine prenatal care, which is entirely covered by publicly funded basic health insurance.

Consecutive pregnant women in the 35–37 weeks of pregnancy were included. After an informed consent to participate in a study was obtained, two rectovaginal swabs were obtained from each participant with the same technique except that, swab A was taken by HCWs using a sterile UNI-TER swab with Amies transport medium (Meus, Piove di Sacco, Italy), which is currently the standard method of GBS prenatal screening in Slovenia; swab B was collected by the pregnant woman herself using a sterile FLOQSwab with LIM broth enrichment transport medium (Copan, Brescia, Italy) according to the visual instructions (Supplementary Figure S1) in a separate room without the presence of the HCW at the end of the same office visit. Both swabs were transported and plated the same day in the microbiology laboratory. After sampling, all study participants received an anonymous and de-identified questionnaire with additional demographic and self-assessment questions (Supplementary Table S1).

The study was conducted in accordance with the Declaration of Helsinki, and the protocol was approved by the Ethics Committee of the Ministry of Health of the Republic of Slovenia (No. 0120-98/2023/5; 30.5.2023). The study was not registered on EU Clinical Trials Register or ClinicalTrials.org.

### 2.2 Microbiological methods and testing algorithm

Laboratory analyses were performed at the National Laboratory of Health, Environment and Food, Novo mesto, Slovenia. Upon arrival at the laboratory, both swabs A and B were first plated directly onto ChromID Strepto B (STRB) agar (bioMérieux, Marcy-l’Etoile, France) using the standard manual four-quadrant streaking method. Swab A was then used to inoculate 2 mL of in-house LIM broth prepared from dehydrated Todd-Hewitt broth (Oxoid, Basingstoke, United Kingdom) supplemented with colistin (10 μg/mL) and nalidixic acid (15 μg/mL) (Oxoid, Basingstoke, United Kingdom), while swab B was already in the original LIM broth (Copan, Brescia, Italy). Both LIM broths were mechanically shaken for 5–10 s to release the bacteria from the tip of the swab and incubated overnight in an aerobic atmosphere without the swab for at least 18 h (Verani et al., 2010).

On the day 2, 100 μL of the LIM broth was inoculated to the second STRB agar for a further 48-h incubation (Rosa-Fraile and Spellerberg, 2017). During the incubation, all agar plates, i.e., those inoculated directly and those inoculated after enrichment, were examined daily for the appearance of typical pale pink to red colonies, all of which were confirmed for identification using the MALDI Biotyper Compass, Library revision K (Bruker Daltonics, Bremen, Germany).

Molecular detection of GBS was performed on the second day from the enriched LIM broth after at least 18 h of incubation. We used the FDA-approved NeuMoDx GBS assay with the NeuMoDx 96 Molecular System (Qiagen, Hilden, Germany). This fully automated, random access molecular system combines automated nucleic acid extraction, amplification and detection with Taqman chemistry and an advanced microfluidic cartridge design (Emery et al., 2019). The NeuMoDx GBS assay amplifies an 88 bp fragment of the *pcsB* gene from the GBS chromosome and uses 25 μL of LIM broth as a starting sample volume. The laboratory workflow diagram is presented in Supplementary Figure S2.

In case of repetitive discrepant results between culture and NeuMoDx GBS assay, the discrepancy was resolved with a second molecular assay, the Xpert GBS assay (Cepheid, Sunnyvale, CA, United States), which amplifies and detects the *cfb* gene of GBS, i.e., a different molecular target than the NeuMoDx GBS assay. The Xpert GBS assay was performed with 200 μL of LIM broth added to the sample chamber of the test cartridge (Buchan et al., 2015).

Samples that were (a) positive by culture and NeuMoDx GBS assay (cycle threshold, Ct <35) or (b) negative by culture but positive by both molecular assays (both Ct <35) were classified as true positive for the presence of GBS, and this result was used as a reference standard for calculating test performance and sample collection performance (Tables 3, 2). Index and reference test results were available to the obstetrician on request. The laboratory staff were unaware of the clinical information when the test was performed. If one of the tests were positive, the IAP was administered during delivery self-assessment questionnaire.

**Table 1:** Demographic characteristics and questionnaire-based data.

Patients	Number	Proportion	
	*n*	%	(95% CI)
All	227	100	/
Mean age	30.8 (30.2 to 31.4)	/	/
Minimum age	20	/	/
Maximum age	44	/	/
Site ^a^			
Site A	136	59.9	/
Site B	48	21.1	/
Site C	43	18.9	/
Education (*n* = 218)^b^			
Primary school	1	0.5	(0.1–2.6)
Secondary school	67	30.7	(25.0–37.1)
University	145	66.5	(60.0–72.4)
Doctoral degree	5	2.3	(1.0–5.3)
Previous pregnancies (*n* = 218)^b^			
0	95	43.6	(37.2–50.2)
1	69	31.7	(25.8–38.1)
2	29	13.3	(9.4–18.5)
≥3	25	11.5	(7.9–16.4)
Acceptability of self-collection (*n* = 219)^b^			
1 (acceptable)	6	2.7	(1.3–5.9)
2	35	16.0	(11.7–21.4)
3	44	20.1	(15.3–25.9)
4	67	30.6	(24.9–37.0)
5 (unacceptable)	67	30.6	(24.9–37.0)
Simplicity of self-collection (*n* = 219)^b^			
1 (simple)	3	1.4	(0.5–3.9)
2	15	6.8	(4.2–11.3)
3	27	12.3	(8.6–17.3)
4	59	26.9	(21.5–33.2)
5 (difficult)	115	52.5	(45.9–59.0)
Preference (*n* = 212)^b^			
HCW-collection	124	58.5	(51.8–64.9)
Self-collection	75	35.4	(29.3–42.0)
No preference	13	6.1	(3.6–10.5)

a Site A University Medical Center Ljubljana, Site B Novo mesto Community Health Center, Site C Trebnje Community Health Center.

b Questionnaire-based data, the number of responses is shown in brackets.

**Table 2:** Performance of GBS detection with direct culture, enrichment culture and enrichment PCR using two collection strategies.

Method	Performance			Contingency tables						
		HCW	Self		HCW			Self		
Direct culture (24 h)^a^	Sensitivity	69.8	76.7		Pos	Neg	Total	Pos	Neg	Total
	Specificity	100.0	100.0	TP	30	13	43	33	10	43
	PPV	100.0	100.0	TN	0	184	184	0	184	184
	NPV	93.4	94.9	Total	30	197	227	33	194	227
Direct culture (48 h)	Sensitivity	83.7	90.7		Pos	Neg	Total	Pos	Neg	Total
	Specificity	100.0	100.0	TP	36	7	43	39	4	43
	PPV	100.0	100.0	TN	0	184	184	0	184	184
	NPV	96.3	97.9	Total	36	191	227	39	188	227
Enrichment culture (48 h)	Sensitivity	83.7	86.1		Pos	Neg	Total	Pos	Neg	Total
	Specificity	100.0	100.0	TP	36	7	43	37	6	43
	PPV	100.0	100.0	TN	0	184	184	0	184	184
	NPV	96.3	96.8	Total	36	191	227	37	190	227
Enrichment culture (72 h)	Sensitivity	86.1	86.1		Pos	Neg	Total	Pos	Neg	Total
	Specificity	100.0	100.0	TP	37	6	43	37	6	43
	PPV	100.0	100.0	TN	0	184	184	0	184	184
	NPV	96.8	96.8	Total	37	190	227	37	190	227
Enrichment PCR (24 h)	Sensitivity	93.0	100.0		Pos	Neg	Total	Pos	Neg	Total
	Specificity	100.0	100.0	TP	40	3	43	43	0	43
	PPV	100.0	100.0	TN	0	184	184	0	184	184
	NPV	98.4	100.0	Total	40	187	227	43	184	227

HCW, healthcare-worker-collection protocol; self, self-collection protocol; PPV, positive predictive value; NPV, negative predictive value; Pos, positive; Neg, negative; TP, true positive [samples positive by (a) culture and NeuMoDx GBS assay or (b) negative by culture but positive by both molecular assays; NeuMoDx GBS assay and GXpert GBS assays]; TN, true negative.

a Hours in brackets represent time from collection of sample to result.

**Table 3:** Comparison between effectiveness of colonization detection using two collection strategies and different detection algorithms included in the study.

		Self-collection^a^				
		Δ *n* (*p*-value)				
		Culture (24 h)	Culture (48 h)	Enrichment culture (48 h)	Enrichment culture (72 h)	Enrichment PCR (24 h)
HCW-collection	Culture (24 h)	+3 (0.083)	+9 (0.003)	+7 (0.008)	+7 (0.008)	+13 (<0.001)
	Culture (48 h)	−3 (0.180)	+3 (0.083)	+1 (0.317)	+1 (0.317)	+7 (0.008)
	Enrichment culture (48 h)	−3 (0.180)	+3 (0.083)	+1 (0.317)	+1 (0.317)	+7 (0.008)
	Enrichment culture (72 h)	−4 (0.102)	+2 (0.317)	0 (nd)	0 (nd)	+6 (0.014)
	Enrichment PCR (24 h)	−7 (0.008)	−1 (0.317)	−3 (0.083)	−3 (0.083)	+3 (0.083)

a Difference (Δ) in a number of positives is expressed as positives following self-collection protocol subtracted by positives following HCW collection protocol. Significance is calculated with McNemar’s test.

### 2.3 Self-assesment questionnaire

Participants completed a self-assessment questionnaire after sampling (Supplementary Table S1) to provide structured feedback on self-collection. Data collected included demographics (e.g., age, pregnancies, education, income), self-collection performance difficulty, acceptability versus standard collection, and participant preferences. Questionnaire data were not linked to test results.

### 2.4 Statistical analysis

Descriptive statistics were used for the demographic parameters of the sample. Data were presented with proportions and 95% confidence intervals. Performance characteristics were calculated for combinations of sampling method and diagnostic test algorithm. Positive and negative predictive values were calculated based on the prevalence of colonization in the sample. The McNemar’s test was used to compare different methods within the paired samples. Statistical analyses was performed using the statistical package JASP (version 0.19) (JASP Team, 2024).

A sample size of at least *n* = 221 paired measurements was set to achieve a power of 80% with a two-sided significance of 5%, assuming that up to 3% of pairs change from positive to negative and 10% from negative to positive, i.e., to detect a difference of 0.07 between the discordant proportions, values expected from previous comparative studies (Dhand and Khatkar, 2014).

## 3 Results

A total of 227 pregnant women (mean age 30.8 years; range 20 to 44) from the UMC Ljubljana (*n* = 136), the CHC Novo mesto (*n* = 48) and the CHC Trebnje (*n* = 43) were included. Most women had a university degree (66.5%) and were nulliparous (43.6%). The demographic data are presented in Table 1.

Overall, GBS was detected in 18% (95% CI 13–23%; *n* = 40) of swabs A and 19% (95% CI 14–25%; *n* = 43) of swabs B. Three additional colonized women were detected in the self-collected group by either method. Table 2 shows the performance characteristics of detection methods within two groups of swab collection. Briefly, in the HCW-collected swabs, direct culture on selective STRB agar had a sensitivity of 69.8 and 83.7% after one and two days of incubation, respectively. This was only slightly less than the sensitivity of the enrichment culture, which was 83.7 and 86.1% after one- and two-day incubation, respectively (Table 2). A similar pattern was observed in self-collected swabs, where the additional enrichment step was not associated with better performance characteristics. In fact, the sensitivity of the direct culture showed a trend towards higher sensitivity after 2 days of incubation compared to the enrichment culture (90.7% vs. 86.1%, *p* = 0.317).

The PCR results were superior to both culture methods in both groups. In the group of swabs collected by HCWs, 4 (40 vs. 36; 11.1% difference; *p* = 0.046) and 3 (40 vs. 37; 8.1% difference; *p* = 0.083) additional positives were detected with PCR compared to direct and enrichment culture, respectively; in the group of self-collected swabs, 4 (43 vs. 39; 10.3% difference; *p* = 0.046) and 6 (43 vs. 37; 16.2% difference; *p* = 0.014) additional positives were detected with PCR compared to direct and enrichment culture, respectively. For samples collected by HCWs, the difference in sensitivity was 93.0% vs. 83.7% (*p* = 0.046) for direct culture and 93.0% vs. 86.1% (*p* = 0.083) for enrichment culture as compared to PCR. For the self-collected samples, the difference in sensitivity reached statistical significance for both the direct culture (100.0% vs. 90.7%; *p* = 0.046) and the enrichment culture (100.0% vs. 86.1%; *p* = 0.014).

Table 3 shows the comparison of the different detection methods between HCW-collected and self-collected samples. As observed, self-collection showed a trend towards a higher diagnostic yield in head-to-head comparisons of all diagnostic methods, albeit without statistical significance (diagonal in Table 3). Nevertheless, PCR after enrichment from self-collected samples was the most sensitive method overall and reached statistical significance for all detection methods, except for PCR from HCW-collected samples. A difference of 16.2% (43 vs. 37) was observed when comparing the PCR of self-collected swabs with the enrichment culture collected by the physician, a current reference standard in Slovenia.

Specificity was 100% both for culture and PCR detection. For culture, this was because we confirmed identification of all typical colonies with MALDI-TOF. Altogether, 13 women in both types of samples grew suspicious pale pink to red colonies that were identified as *Streptococcus oralis* (*n* = 10), *Streptococcus parasanguinis* (*n* = 2) and *Streptococcus salivarius* (*n* = 1). As for the PCR detection, all NeuMoDx positive samples were confirmed to be positive also with the confirmatory GenXpert GBS PCR.

Although majority of women found self-collection of swabs simple (79.4%) and acceptable (61.2%), 58.5% (*n* = 124/212; 52–65%) of pregnant women would prefer the collection of rectovaginal swabs by HCWs.

## 4 Discussion

GBS is a leading cause of early neonatal sepsis, largely preventable through active screening and IAP. The typically low bacterial load in colonized pregnant women highlights the need for high detection sensitivity, as most invasive infections, after the introduction of screening programs, occur in women with negative screening results (Dyke et al., 2009; Mirsky et al., 2020). In Slovenia, one of the most important improvements in prevention was the introduction of universal GBS screening with PCR after enrichment at 35–37 weeks of pregnancy. The rectovaginal swab is taken as standard by HCWs, mostly by gynecologists using traditional swabs with semi-solid transport media. In our study, we tested whether self-collection with the commercial LIM broth flocked swabs using a fully automated FDA-approved PCR test could improve detection. We have shown that improvements in pre-analytical and analytical processing are possible in our clinical setting. In addition, our results emphasize that swab collection must be quality-assured, regardless of whether it is performed by medical personnel or by a self-collection protocol. We have also shown that commercial LIM broths and flocked swabs may be a better collection tool compared to standard swabs, albeit possibly slightly higher price. We also confirmed that PCR from enrichment broth is more sensitive than direct culture or enrichment culture, although direct culture using LIM broth and flocked swabs instead of standard swabs was also excellent.

Sample collection by medical personnel offers several advantages, such as better access to vaginal and rectal sites and visual control. However, for some pregnant women, swabs collection by a doctor may be perceived as unpleasant or intrusive and cause discomfort. Self-collection could be an alternative that increases patient engagement with screening and allows healthcare providers to spend more time on other tasks during the office visit. In our study, self-collection provided better results than the control group.

Several other studies have compared these two strategies in different clinical settings. As early as 1995, Mercer et al. (1995) reported higher detection of GBS when patients collected their own separate vaginal and anal swabs, with a combined sensitivity of 91.7% versus 70.8% in favor of self-collection. Hicks and Diaz-Perez (2009) compared the results of GBS cultures in community health centers with self-collection and physician-collection. The self-collection centers had a higher positivity rate (13.3%) than the physician-collection centers (10.6%), and the study was conducted in a predominantly Hispanic, lower socioeconomic population. Our results are in line with findings of these studies indicating a higher diagnostic accuracy when samples are collected by pregnant women themselves as compared to HCWs.

Some studies, however, yielded contrasting results compared to the present study. Torok and Dunn (2000) reported higher positivity in rectovaginal swabs collected by a physician compared to self-collected swabs, but with no statistically significant difference. Similarly, a 2006 Canadian study reported a higher GBS colonization prevalence in the physician-collected swab group of 18.8% compared to 17.0% in the self-collection swab group and sensitivities of 96.9 and 87.5%, respectively (Price et al., 2006). In this study, 27.3% of pregnant participants preferred self-collection, 56.3% had no preference and 22.7% preferred collection by a service provider. The same pattern was found in an Irish study by Arya et al. (2008). GBS prevalence was 11.7% in the group collected by healthcare professionals versus 9.8% in the self-collected group, with a sensitivity of 97.5% versus 94.3%. The agreement of the results was 97.5% with a kappa value of 0.87, indicating excellent comparability between the groups. Nevertheless, most participants were in favor of collection by medical personnel (Arya et al., 2008).

Finally, in a systematic review and meta-analysis from 2023, the authors summarized the results of 10 studies comparing the concordance of self-collected vaginal and rectal swabs with those collected by healthcare personnel (i.e., the gold standard). The overall sensitivity and specificity of self-collection were 90 and 98%, respectively. The authors concluded that the results of swabs taken by the women themselves for the detection of GBS carriage were comparable to those taken by healthcare professionals (Odubamowo et al., 2023). The American College of Obstetricians and Gynecologists (ACOG) does not directly recommend self-collection but states, that pregnant women can take a rectovaginal swab themselves if they are given clear and understandable instructions (The American College of Obstetricians and Gynecologists, 2020). The varying results of studies on the accuracy and acceptability of self-collection for GBS detection indicate that these findings cannot be universally applied, as they differ significantly across different groups of pregnant women. Therefore, it is crucial to assess whether self-collection is both reliable and acceptable for GBS screening in each specific population before adopting this method.

Molecular methods have significantly improved diagnostic microbiology. There are numerous FDA-approved molecular tests for the detection of GBS colonization in pregnant women, although most require a prior enrichment step for accurate GBS confirmation (Perme et al., 2021). Only one test currently supports direct detection during labor. Enrichment is essential for detection, as the bacterial load of GBS in colonized women may be low. Molecular GBS detection after overnight enrichment in a liquid culture improves sensitivity and shortens test duration. In several previous studies, 20–40% more cases of colonization were identified within 24 h than with the standard CDC method, which represents an advantage in terms of sensitivity and time to detection of 1–2 days compared to conventional methods (Couturier et al., 2014; Miller et al., 2015; Hernandez et al., 2018; Shin and Pride, 2019; Emery et al., 2019). Therefore, the combination of enrichment and molecular detection is increasingly seen as a potential new gold standard for GBS screening (Rosa-Fraile and Spellerberg, 2017).

In our study, we used chromogenic agar that was inoculated directly and after enrichment. The main advantage of chromogenic agars is the ability to detect both non-hemolytic and non-pigmented GBS strains, which are present in 2–5% of positive samples (Rosa-Fraile and Spellerberg, 2017). We found a minimal advantage between enrichment culture on day 2 after sample collection and direct culture on day 2 after 48 h of incubation. This is most likely due to better color development over a longer period of time and the lack of overgrowth with enterococci in the case of lower GBS colonization. However, on day 2, several suspicious colonies had to be properly identified by MALDI-TOF due to the light red to pink color development, most of which turned out to be other *Streptococcus* species (Rosa-Fraile and Spellerberg, 2017; Joubrel et al., 2014).

We used a combination of flocked swabs and liquid enrichment broth as a transport medium for self-collected swabs. To the best of our knowledge, there are no studies evaluating this combination for self-collection, however, a combination of flocked swabs and a liquid transport medium has been shown to increase the recovery of bacteria from clinical sites compared to standard fiber-coated swabs used in the control group (Silbert et al., 2016; Nys et al., 2010).

Our study has several limitations. (i) Although we have achieved statistical power, the small sample size makes it difficult to generalize the results to the broader population, which would require larger studies. (ii) The study was pragmatic in nature, and the two testing algorithms differed in several parameters that could influence the recovery and detection of GBS, particularly the collection device. (iii) For molecular detection, we used the NeuMoDx system, which at the time of testing was one of the most promising PCR systems for mid-sized laboratories as it was fully automated, allowed random access, and performed FDA-approved tests, but at the time of writing this system has been discontinued. The molecular results can therefore only be used as a general concept. (iv) We did not specifically ask about antibiotic consumption among participants, which could be a potential factor contributing to false-negative culture results. (v) We could not link the test results and the questionnaire results individually for ethical reasons, so we could only analyze the questionnaire as a group.

The results of our study have reinforced the case for molecular testing in the GBS screening programs. In addition, we have gathered solid data supporting both self-collection for screening and the use of commercial collection-enrichment device such as the one tested here, which have also proven to be practical in the laboratory.

## 5 Conclusion

Self-collection of rectovaginal swabs during pregnancy is a valid alternative to swabs taken by medical personnel. PCR was better than enrichment culture for the detection of GBS. PCR results were available within 24 h. Nevertheless, the majority women preferred swabs taken by HCWs. Commercial liquid-based enrichment swabs simplified laboratory workflows, but their impact on results could not be determined in this study design. Quality-assured sample collection is of great importance for both HCW-collected and self-collection of rectovaginal swabs for the detection of GBS colonization.

## Data Availability

The raw data supporting the conclusions of this article will be made available by the authors, without undue reservation.

## References

[ref1] Arbyn M. Verdoodt F. Snijders P. J. F. Verhoef V. M. J. Suonio E. Dillner L.. (2014). Accuracy of human papillomavirus testing on self-collected versus clinician-collected samples: a meta-analysis. Lancet Oncol. 15, 172–183. doi: 10.1016/S1470-2045(13)70570-9 24433684

[ref2] Arya A. Cryan B. O’Sullivan K. Greene R. A. Higgins J. R. (2008). Self-collected versus health professional-collected genital swabs to identify the prevalence of group B streptococcus: a comparison of patient preference and efficacy. Eur. J. Obstet. Gynecol. Reprod. Biol. 139, 43–45. doi: 10.1016/j.ejogrb.2007.12.005, PMID: 18255214

[ref3] Boyer K. M. Gadzala C. A. Kelly P. D. Burd L. I. Gotoff S. P. (1983). Selective intrapartum chemoprophylaxis of neonatal group B streptococcal early-onset disease. II. Predictive value of prenatal cultures. J. Infect. Dis. 148, 802–809. doi: 10.1093/infdis/148.5.802, PMID: 6355317

[ref4] Boyer K. M. Gotoff S. P. (1986). Prevention of early-onset neonatal group B streptococcal disease with selective intrapartum chemoprophylaxis. N. Engl. J. Med. 314, 1665–1669. doi: 10.1056/NEJM198606263142603, PMID: 3520319

[ref5] Buchan B. W. Faron M. L. Fuller D. Davis T. E. Mayne D. Ledeboer N. A. (2015). Multicenter clinical evaluation of the Xpert GBS LB assay for detection of group B streptococcus in prenatal screening specimens. J. Clin. Microbiol. 53, 443–448. doi: 10.1128/JCM.02598-14, PMID: 25411176 PMC4298547

[ref6] Cortese F. Scicchitano P. Gesualdo M. Filaninno A. De Giorgi E. Schettini F.. (2016). Early and late infections in newborns: where do we stand? A review. Pediatr. Neonatol. 57, 265–273. doi: 10.1016/j.pedneo.2015.09.007, PMID: 26750406

[ref7] Couturier B. A. Weight T. Elmer H. Schlaberg R. (2014). Antepartum screening for group B streptococcus by three FDA-cleared molecular tests and effect of shortened enrichment culture on molecular detection rates. J. Clin. Microbiol. 52, 3429–3432. doi: 10.1128/JCM.01081-14, PMID: 25009049 PMC4313176

[ref8] Dhand N. K. Khatkar M. S.. (2014). Statulator: an online statistical calculator. Sample size calculator for comparing two paired proportions. Available at: http://statulator.com/SampleSize/ss2PP.html. (Accessed November 2, 2024)

[ref9] Dyke M. K. V. Thomas A. R. Mohle-Boetani J. Petit S. Spina N. L. Shutt K. A.. (2009). Evaluation of universal antenatal screening for group B streptococcus. N. Engl. J. Med. 360, 2626–2636. doi: 10.1056/NEJMoa0806820 19535801

[ref10] Edmond K. M. Kortsalioudaki C. Scott S. Schrag S. J. Zaidi A. K. Cousens S.. (2012). Group B streptococcal disease in infants aged younger than 3 months: systematic review and meta-analysis. Lancet 379, 547–556. doi: 10.1016/S0140-6736(11)61651-6 22226047

[ref11] Emery C. L. Relich R. F. Davis T. H. Young S. A. Sims M. D. Boyanton B. L. (2019). Multicenter evaluation of NeuMoDx group B streptococcus assay on the NeuMoDx 288 molecular system. J. Clin. Microbiol. 57, e01324–e01318. doi: 10.1128/JCM.01324-18, PMID: 30463895 PMC6355512

[ref12] Hernandez D. R. Wolk D. M. Walker K. L. Young S. Dunn R. Dunbar S. A.. (2018). Multicenter diagnostic accuracy evaluation of the Luminex Aries real-time pcr assay for group B streptococcus detection in LIM broth-enriched samples. J. Clin. Microbiol. 56:e01768. doi: 10.1128/JCM.01768-17, PMID: 29848562 PMC6062795

[ref13] Hicks P. Diaz-Perez M. J. (2009). Patient self-collection of group B streptococcal specimens during pregnancy. J. Am. Board Fam. Med. 22, 136–140. doi: 10.3122/jabfm.2009.02.080011, PMID: 19264936

[ref14] JASP Team (2024). JASP (version 0.19.0). Amsterdam: JASP.

[ref15] Joubrel C. Gendron N. Dmytruk N. Touak G. Verlaguet M. Poyart C.. (2014). Comparative evaluation of 5 different selective media for group B streptococcus screening in pregnant women. Diagn. Microbiol. Infect. Dis. 80, 282–284. doi: 10.1016/j.diagmicrobio.2014.08.005, PMID: 25249270

[ref16] Lasič M. Lučovnik M. Pavčnik M. Kaparič T. Ciringer M. Lozar Krivec J.. (2018). Invasive neonatal *Streptococcus agalactiae* infection in Slovenia, 2003–2013. Slov. Med. J. 86, 493–506. doi: 10.6016/ZdravVestn.2464, PMID: 23086009

[ref17] Lučovnik M. Tul Mandić N. Lozar Krivec J. Dolinar U. Jeverica S. (2016). Prevalence of *Streptococcus agalactiae* colonisation among pregnant women in Slovenia, 2013–2014. Slov. Med. J. 85, 393–400. doi: 10.6016/ZdravVestn.1373, PMID: 23086009

[ref18] Madrid L. Seale A. C. Kohli-Lynch M. Edmond K. M. Lawn J. E. Heath P. T.. (2017). Infant group B streptococcal disease incidence and serotypes worldwide: systematic review and meta-analyses. Clin. Infect. Dis. 65, S160–S172. doi: 10.1093/cid/cix656, PMID: 29117326 PMC5850457

[ref19] Mercer B. M. Taylor M. C. Fricke J. L. Baselski V. S. Sibai B. M. (1995). The accuracy and patient preference for self-collected group B streptococcus cultures. Am. J. Obstet. Gynecol. 173, 1325–1328. doi: 10.1016/0002-9378(95)91380-7, PMID: 7485347

[ref20] Miller S. A. Deak E. Humphries R. (2015). Comparison of the AmpliVue, BD Max System, and *illumi* gene molecular assays for detection of group B streptococcus in antenatal screening specimens. J. Clin. Microbiol. 53, 1938–1941. doi: 10.1128/JCM.00261-15, PMID: 25788551 PMC4432075

[ref21] Mirsky R. Carpenter D. M. Postlethwaite D. A. Regenstein A. C. (2020). Preventing early-onset group B streptococcal sepsis: is there a role for rescreening near term? J. Matern. Fetal Neonatal Med. 33, 3791–3797. doi: 10.1080/14767058.2019.1586874, PMID: 30890002

[ref22] Nys S. Vijgen S. Magerman K. Cartuyvels R. (2010). Comparison of Copan Eswab with the Copan Venturi Transystem for the quantitative survival of *Escherichia coli, Streptococcus agalactiae* and *Candida albicans*. Eur. J. Clin. Microbiol. Infect. Dis. 29, 453–456. doi: 10.1007/s10096-010-0883-5, PMID: 20204445

[ref23] Odubamowo K. Garcia M. Muriithi F. Ogollah R. Daniels J. P. Walker K. F. (2023). Self-collected versus health-care professional taken swab for identification of vaginal-rectal colonisation with group B streptococcus in late pregnancy: a systematic review. Eur. J. Obstet. Gynecol. Reprod. Biol. 286, 95–101. doi: 10.1016/j.ejogrb.2023.05.027, PMID: 37229964

[ref24] Official Gazette of the Republic of Slovenia (2023). Official Gazette of the Republic of Slovenia, No. 39/2023. Ljubljana: Archives of the Republic of Slovenia, 39.

[ref25] Perme T. Golparian D. Unemo M. Jeverica S. (2021). Lack of diagnostic-escape mutants of group B streptococcus in Slovenia. Clin. Microbiol. Infect. 27, 1054–1055. doi: 10.1016/j.cmi.2021.01.022, PMID: 33540114

[ref26] Price D. Shaw E. Howard M. Zazulak J. Waters H. Kaczorowski J. (2006). Self-sampling for group B streptococcus in women 35 to 37 weeks pregnant is accurate and acceptable: a randomized cross-over trial. J. Obstet. Gynaecol. Can. 28, 1083–1088. doi: 10.1016/S1701-2163(16)32337-4, PMID: 17169231

[ref27] Rosa-Fraile M. Spellerberg B. (2017). Reliable detection of group B streptococcus in the clinical laboratory. J. Clin. Microbiol. 55, 2590–2598. doi: 10.1128/JCM.00582-17, PMID: 28659318 PMC5648696

[ref28] Russell N. J. Seale A. C. O’Driscoll M. O’Sullivan C. Bianchi-Jassir F. Gonzalez-Guarin J.. (2017). Maternal colonization with group B streptococcus and serotype distribution worldwide: systematic review and meta-analyses. Clin. Infect. Dis. 65, S100–S111. doi: 10.1093/cid/cix658, PMID: 29117327 PMC5848259

[ref29] Shin J. H. Pride D. T. (2019). Comparison of three nucleic acid amplification tests and culture for detection of group B streptococcus from enrichment broth. J. Clin. Microbiol. 57:e01958. doi: 10.1128/JCM.01958-18, PMID: 30944190 PMC6535594

[ref30] Silbert S. Rocchetti T. T. Gostnell A. Kubasek C. Widen R. (2016). Detection of group B streptococcus directly from collected ESwab samples by use of the BD Max GBS assay. J. Clin. Microbiol. 54, 1660–1663. doi: 10.1128/JCM.00445-16, PMID: 27053670 PMC4879306

[ref31] Smith A. C. Thorpe P. G. Learner E. R. Galloway E. T. Kersh E. N. (2024). At-home specimen self-collection as an additional testing strategy for chlamydia and gonorrhoea: a systematic literature review and meta-analysis. BMJ Glob. Health 9:e015349. doi: 10.1136/bmjgh-2024-015349, PMID: 39191483 PMC11404247

[ref32] The American College of Obstetricians and Gynecologists (2020). Prevention of group B streptococcal early-onset disease in newborns: ACOG Committee Opinion, Number 797. Obstet. Gynecol. 135, e51–e72. doi: 10.1097/AOG.0000000000003668, PMID: 31977795

[ref33] Torok P. G. Dunn J. R. (2000). Self-collection of antepartum anogenital group B streptococcus cultures. J. Am. Board Fam. Med. 13, 107–110. doi: 10.3122/15572625-13-2-107, PMID: 10764191

[ref34] Verani J. R. McGee L. Schrag S. J. Division of Bacterial Diseases, National Center for Immunization and Respiratory Diseases, Centers for Disease Control and Prevention (CDC) (2010). Prevention of perinatal group B streptococcal disease—revised guidelines from CDC, 2010. MMWR Recomm. Rep. 59, 1–36. 21088663

[ref35] World Health Organization. (2024). Implementation of self-care interventions for health and well-being guidance for health systems. Available at: https://iris.who.int/bitstream/handle/10665/378232/9789240094888-eng.pdf?sequence=1. (Accessed January 5, 2025)

