# Supplementary material for: Comparison of self-collected and healthcare worker-collected rectovaginal swabs for group B streptococcus detection in pregnancy using PCR with a commercial collection-enrichment device
Source: Front Microbiol. 2025 Feb 5;16:1541319. doi: 10.3389/fmicb.2025.1541319 (PMC11835839; doi:10.3389/fmicb.2025.1541319)
Supplement: Supplementary file 1 [file Data_Sheet_1.docx]

Supplementary Material

# Supplementary Figures and Tables

## Supplementary Figures

**Supplementary Figure 1.** Instructions for self-collection of a group B streptococcus swab


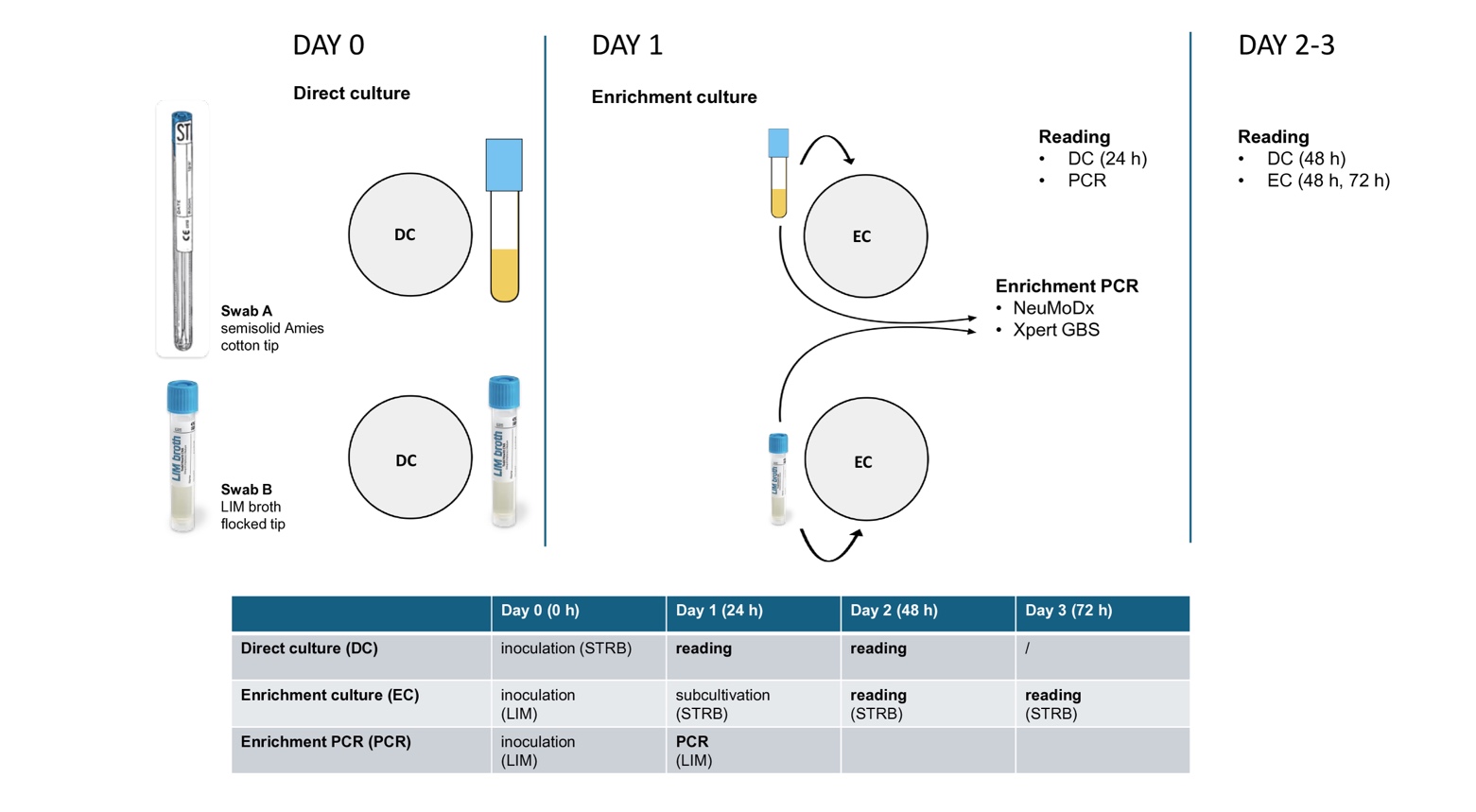


Supplementary Figure 2. Laboratory workflow diagram

## Supplementary Tables

**Supplementary Table 1.** Questionnaire for research participants

**Age: ­­­___________ years**

Number of previous pregnancies: 0 1 2 3 >3

Highest level of education: elementary school high school faculty doctorate

Annual net income (in thousand EUR): <12 >12 >18 >24 >30 >36 >42 >48

**Rate the following statements from 1 to 5**

(1 = strongly disagree, 5 = strongly agree)

| I have enough knowledge about the importance of screening for GBS. | 1 | 2 | 3 | 4 | 5 |
| --- | --- | --- | --- | --- | --- |
| I find self-collection equally acceptable as collection by a nurse/doctor. | 1 | 2 | 3 | 4 | 5 |
| The verbal instructions for self-collection were clear enough. | 1 | 2 | 3 | 4 | 5 |
| The written instructions for self-collection were clear enough. | 1 | 2 | 3 | 4 | 5 |
| The self-collection was easy. | 1 | 2 | 3 | 4 | 5 |
| I had difficulty determining the correct location for the swab collection. | 1 | 2 | 3 | 4 | 5 |
| The self-collection was painful. | 1 | 2 | 3 | 4 | 5 |
| I completely trust the result of self-collection. | 1 | 2 | 3 | 4 | 5 |
| I am concerned that self-collection could harm the fetus. | 1 | 2 | 3 | 4 | 5 |
| I would recommend self-collection to a friend. | 1 | 2 | 3 | 4 | 5 |
| I would also use self-collection for testing other infections. | 1 | 2 | 3 | 4 | 5 |
| Which ones: viral infections, diarrhea, urinary tract infections, vaginosis, sexually transmitted infections, HPV | | | | | |
| Which ones (specify): | | | | | |

**Which type of swab collection would you choose for a future pregnancy?**

self-collection nurse/doctor collection
